# A super-resolution approach for receptive fields estimation of neuronal ensembles

**DOI:** 10.1186/1471-2202-16-S1-P130

**Published:** 2015-12-18

**Authors:** Daniela Pamplona, Gerrit Hilgen, Bruno Cessac, Evelyne Sernagor, Pierre Kornprobst

**Affiliations:** 1 INRIA Sophia Antipolis Méditerranée, Neuromathcomp, 2004 Route des Lucioles, 06902 Valbonne, France; 2 Institute of Neuroscience, Faculty of Medical Sciences, Framlington Place, Newcastle upon Tyne, NE2 4HH

## 

**Figure 1:**
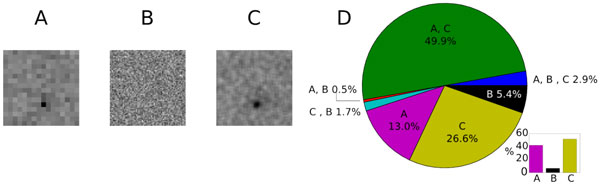
**A, B, C: Representative example of STA of one neuron considering stimulus A, B) and C)**. **D**: Number of RFs mapped with each stimulus.

The Spike Triggered Average (STA) is a classical technique to find a discrete approximation of the Receptive Fields (RFs) of sensory neurons [1], a required analysis in most experimental studies. One important parameter of the STA is the spatial resolution of the estimation, corresponding to the size of the blocks of the checkerboard stimulus images. In general, it is experimentally fixed to reach a compromise: If too small, neuronal responses might be too weak thus leading to RF with low Signal-to-Noise-Ratio; on the contrary, if too large, small RF will be lost, or not described with enough details, because of the coarse approximation. Other solutions were proposed consisting in starting from a small block size and updating it following the neuron response in a closed-loop to increase its response [2-4]. However, these solutions were designed for single cells and cannot be applied to simultaneous recordings of ensembles of neurons (since each RF has its own size and preferred stimulus). To solve this problem, we introduced a modified checkerboard stimulus where blocks are shifted randomly in space at fixed time steps. This idea is inspired from super-resolution techniques developed in image processing [4]. The main interest is that the block size can be large, enabling strong responses, while the resolution can be finer since it depends on the shift minimum size. In [5] was shown that the STA remains an unbiased RF estimator and, using simulated spike trains from an ensemble of Linear Nonlinear Poisson cascade neurons, it was predicted that this approach improves RF estimation over the neuron ensemble. Here, we test these predictions experimentally on the RFs estimation of 8460 ganglion cells from two mouse retinas, using recordings performed with a large scale high-density multielectrode array. To illustrate the main interest of the approach, in Figure 1 we show a representative example of STA for one neuron where RFs have been obtained using the three following stimuli (all presented during 15min, for one retina displayed at 10 Hz, for the other at 30 Hz): (A) standard checkerboard stimulus with block size of 160μm, (B) standard checkerboard stimulus with block size of 40μm, (C) checkerboard stimulus with block size of 160μm and arbitrary shifts of 40μm in × and y-directions. Results show spatial resolution can be improved in case (C), while nothing could be obtained in (B) by changing only the block size of the standard stimulus. At the population level, plot (D) shows the number of the RFs that could be recovered for each stimuli, using a decision criteria based of the RFs value distribution. Most of the RFs were mapped with both methods (A) and (C) (49.9%). However, the proposed case (C) allows to recover 51% of the mapped RFs at a resolution of 40μm, while in the classical case (A), 41% of the RFs could be found at a resolution of only 160μm. Thus, the method does improve the quality of the RF estimation and the amount of successfully mapped RFs in neural ensembles.
